# Palliative care provision for children in general practice: A retrospective cohort study

**DOI:** 10.1017/S1478951526102077

**Published:** 2026-03-26

**Authors:** Marijanne Engel, Matthew P. Grant, Anna Kleiboer, Everlien de Graaf, Saskia C.C.M. Teunissen, Marijke C. Kars

**Affiliations:** 1 Julius Center for Health Sciences and Primary Care, Department of General Practice and Nursing Science, Center of Expertise in Palliative Care Utrecht, University Medical Center Utrecht, Utrecht University, Utrecht, The Netherlands; 2 Department of Internal Medicine, Meander Medical Center, Amersfoort, the Netherlands

**Keywords:** general practice, palliative care, pediatrics, Child, Family, cohort study

## Abstract

**Objectives:**

In the Netherlands, around 750 children (0–21 year) die annually from potentially palliative conditions. The majority of these children reside at home, receiving care from hospital services and primary care. This study aims to examine general practice utilization for pediatric palliative care patients in the last 2 years of life.

**Methods:**

A retrospective cohort study was performed using the routine healthcare database of the Julius General Practitioners’ Network. The main outcome for general practitioner (GP) utilization was the number of GP consultations for children in the last 3 months of life. Participants were included who were children (0–21 years), and deceased in the period 01-01-2013 to 31-12-2022 from an underlying chronic condition. Data were analyzed using descriptive statistics and tested for differences in provided care between children who died in hospital and who died at home.

**Results:**

Forty-eight children from 32 GP practices met inclusion criteria. Median age was 10.0 years (interquartile range [IQR] 1.5–17.1). Common diagnoses were oncological (29%), congenital (29%), and metabolic conditions (23%). Ninety-six percent of children had contact with their GP in the last 3 months (median 7 consultations, IQR 3.0–10.0), i.e. 26 children who died in hospital had median 3.5 GP consultations compared to 20 children who died at home median 9.5 GP consultations (*p* < 0.001). Thirty-five percent of children were documented as being palliative, with 54% having some form of advance care planning discussions documented.

**Significance of results:**

These results demonstrate that GPs are highly involved in providing pediatric palliative care. The palliative nature of these children and advance care planning discussions are not routinely documented and/or performed by GPs. Further insights into guidance that supports GPs, in collaboration with other healthcare professionals, in providing palliative care for children at home and their families are needed.

## Introduction

In the Netherlands, around 750 children (0–21 year) die annually from potentially palliative conditions (CBS StatLine 2024). Potentially palliative conditions refer to a spectrum of actual or potentially life-limiting and life-threatening conditions in children (malignant and non-malignant), for some of which curative treatment may be feasible, but where that treatment may also fail. Examples are oncological, congenital, neurological, metabolic, and cardiovascular conditions (Fraser et al. 2020). Due to improved treatment options and symptom management, the majority of children with serious illness reside and access care in the home setting. These children receive medical care through specialist pediatricians and general practitioners (GPs) (Vallianatos et al. 2021).

In the Netherlands, every Dutch citizen is registered in a GP practice that offers person-focused medical care over time to registered patients (Stegeman et al. 2025). In general, in case of medical problems, for patients of all ages the GP is the first point of contact and will refer to other healthcare professionals if necessary. In many Western countries including the Netherlands, GPs play also a vital role in providing out-of-hours primary care (Bomholt et al. 2024).

It is known, that GPs fulfil a pivotol role in identifying when patients may have palliative care needs and providing this care for adult patients (Johnson et al. 2018; Van der Plas et al. 2018; Rhee et al. 2020; Engel et al. 2022; Grant et al. 2024b). However, pediatric palliative care differs from palliative care for adults for several reasons: children in need of palliative care often suffer from different diseases than adults, and these may be rare, complex diseases, with many different symptoms (Straatman and Miller 2013; Vallianatos et al. 2021). In the Netherlands, because of the complex nature, pediatric palliative care has been developed primarily in the seven university hospitals and the specialized Center for Pediatric Oncology (Vallianatos et al. 2021). Due to the complexity of this care, the care is often lead by a specialist pediatrician, who is often affiliated with one of these university or specialized hospitals. In order to contribute to the best possible quality of life for children and their families, pediatric palliative care is shifting toward the home situation, increasing the importance of the specific role of the GP and other primary care professionals, and collaborations between generalist and specialist healthcare professionals (Abel et al. 2018; Vallianatos et al. 2021; Schröder et al. 2024; Skjærseth et al. 2025). While guidelines recommend that GPs become involved earlier and may take the leading role in palliative care at home, these exact roles in care provision of GPs and pediatricians differ substantially between pediatric palliative care patients, likely reflecting the heterogenous patient population, skills and experience of healthcare professionals, and diversity of patient care needs and those of the family (NICE Guideline 2019; Vallianatos et al. 2021; Dutch Association for Pediatrics [NVK] 2022). The role of the GP as family physician allows active identification of the family’s needs for support during the care process. This includes advance care planning (ACP), symptom management for the child, grief support for the child and family during the child’s life, and bereavement care for the family after death (Dutch Association for Pediatrics [NVK] 2022).

In a systematic review on GP and GP nurse participation in end-of-life care, Rhee et al. (2020) described facilitators and barriers for GP involvement in many areas: patient factors, personal GP factors, GP practice factors, relational factors, factors related to the organization of GP care at practice, local and national level (Rhee et al. 2020). For example, in both this systematic review and other studies on palliative care for adult patients several deficits in communication and information exchange between hospital physicians and GPs were identified that may hinder GPs in providing high-quality palliative care (Flierman et al. 2020; Killackey et al. 2020; Rhee et al. 2020; Engel et al. 2022). GPs mentioned poor renumeration as a barrier for providing often time-consuming palliative care (Rhee et al. 2020). In the Netherlands, there is no clear imbursement scheme for palliative care that is provided in collaboration by both hospital services and primary care (Pereira et al. 2024).

Literature demonstrates that GP involvement in pediatric palliative care is challenging (Straatman and Miller 2013; Van der Geest et al. 2017; Mitchell et al. 2021; Dutch Association for Pediatrics (NVK) 2022). In a recent literature study that guided the development of the Dutch pediatric palliative care guidelines (Dutch Association for Pediatrics [NVK] 2022), it was described that children with life-limiting and life-threatening conditions and their families often had poor experiences with GP palliative care (Mitchell et al. 2021). However, both children and family members expressed a wish to receive more support from their GP practice (Mitchell et al. 2021). In a survey study in 2013 among pediatricians and GPs in the province of British Columbia, only 33% of 43 GPs who responded to the survey indicated that their current medical knowledge and experience were sufficient to meet the needs of their pediatric palliative care patients (Straatman and Miller 2013). A striking finding in a study among Dutch GPs on their perspective on home-based palliative care to children with incurable cancer was that in general, GPs reported being satisfied with the quality of the palliative care provided to their pediatric cancer patients, however, they also reported feelings of sadness, helplessness, and stress (Van der Geest et al. 2017).

Although national guidelines emphasize the importance of GPs’ role in pediatric palliative care (Dutch Association for Pediatrics [NVK] 2022), in general, there is a lack of data regarding to what extent GPs actually are involved in pediatric palliative care. It could be assumed that for children for whom home is the (preferred) place of death, GP care provision in the last phase of life is more intensive and timely than for children for whom hospital or, for example, a hospice is the (preferred) place of death, but we could not find any previous studies on this. The aim of this study was to examine to what extent pediatric palliative care patients access GP services and the intensity and timeliness of care provision in the last 2 years of life.

## Methods

### Design and study population

A retrospective cohort study was performed between 9 October 2023 and 31 January 2024. Data were obtained from the Julius General Practitioners’ Network (JGPN) database, which contains routine healthcare data anonymously extracted from electronic primary care settings in the area of Utrecht in the Netherlands (Smeets et al. 2018). This study is reported according to the RECORD statement, a checklist of items that should be reported in observational studies using routinely collected health data (Benchimol et al. 2015).

The initial data were collected from GP records from children who had deceased in the period 01-01-2013 to 31-12-2022, were between 0 and 21 years old and were active patients of a JGPN practice at the time of death. Following inclusion criteria were: death had to be validated in the data and the child had had any form of chronic illness or health issues. Exclusion criterion was if death was due to an acute and unexpected cause (i.e., car accident).

### Database

Data were obtained from the JGPN database (UMC Utrecht: JGPN). The JGPN is a network of around 70 general practices, 200 GPs and 450,000 patients in the region of Utrecht, the Netherlands (UMC Utrecht: JGPN). This database contains all data from routine primary care as documented by affiliated GPs. These include pseudonomized patient characteristics as well as structured data such as International Classification of Primary Care (ICPC)-codes (i.e., symptoms and diagnoses) (WHO Collaborating Centre for the Family of International Classifications [FIC] in the Netherlands), Anatomical Therapeutic Chemical (ATC) Classification (medication) prescriptions (WHO Collaborating Centre for Dug Statistics Methodology) and results of examinations (physical examinations, laboratory tests). The data registered by GPs in free text fields follow the SOAP-Method (Subjective, Objective, Assessment, Plan) describing the content of GP’s consultations.

Data were obtained by pre-selecting, in a limited data set, children with an ICPC-code for “deceased,” a registered date of death in the selected period or information in an open text field related to dying or palliative care. For several children, the ICPC-code for death was missing in the GP’s record, but their records appeared in the first limited data set, for example because in open text fields words possibly related to dying or palliative care were documented (e.g., the child had been prescribed opioids) or the child had been deregistered from the GP practice. From the remaining data set, including data on “sure” and “not sure” deceased children, additional data were requested. These more extensive record data contained coded patient data and GP documentation from the final two years before the date of death. These additional record data were manually screened (AK) on actual death of the child and if so, the cause of death.

### Main outcome

The main outcome for GP utilization was the number of GP consultations for children in the last three months of life. GP consultations were defined as episode of care provision, which involved the GP, and were not purely administrative. Secondary outcome was GP utilization for the included children in the last 2 years of life.

### Description of GP utilization

GP utilization included information on (1) Identification: was the child noted to have palliative intent of treatment. This refers to patients who had palliative needs, where the focus of their treatment was on symptom control and maximizing quality of life. (2) ACP conversations: Any information related to conversations on values, goals, and preferences for treatment and care with child and/or family (Rietjens et al. 2017). Any information related to ACP was considered a component of ACP, even if this was the only time the topic was documented and information on the process of ACP was limited. (3) Symptoms: All documented symptoms at 3 time points: 3 months before death, 1 month before death and 3 days before death. (4) Hospital use and use of out-of-hours service in the last 3 months before death. (5) (Anticipatory) medication used in the last 3 months before death. Anticipatory medications were defined as the proactive prescribing of injectable medicines that are commonly required to control symptoms in palliative care, based on existing literature (Grant et al. 2024a). (6) Terminal phase: The period of inexorable and irreversible decline in functional status before death. This may unfold gradually over days with a fluctuating but nonetheless ongoing decline in a progressive illness, precipitously following an unexpected and devastating neurological event such as a stroke, or following a planned withdrawal of life- sustaining interventions, such as hemodialysis or ventilatory support (Dutch Institute for Palliative Care (PZNL) 2023).

### Data collection

Data was collected for all GP utilization for the included children in the last 2 years of life. We developed a questionnaire for the data collection based on an existing questionnaire designed for a similar mapping study for the adult population (results have not yet been published). The questionnaire was adapted for pediatric palliative care in consultation with three different researchers with clinical backgrounds experienced in (pediatric) palliative care, including the main researcher from the original adult study. A detailed manual was developed to ensure that two or more researchers would register the same data in answers to the questions in the questionnaire. AK collected the data supervised by two experienced researchers. In a weekly researcher group meeting questions on data collection for specific cases were discussed until consensus was reached.

Data were collected on child characteristics, date and location of death, clinical aspects, involved healthcare professionals, documentation of ACP conversations and the use of healthcare services. Data were collected in a cloud-based clinical data management system (Castor Electronic Data Capture [EDC]). The questionnaire is presented in Supplemental file 1.

### Data analysis

Data were analyzed using the statistical program IBM SPSS Statistics (version 29). The results are mainly presented utilizing descriptive statistics. Data were tested for differences in provided GP care between children who died in hospital and who died at home with Mann–Whitney Test and Pearsons’ chi-square test, as appropriate. Text from open text fields was categorized according to the framework of the Dutch Individual Care Plan in pediatric palliative care (Joren et al. 2023).

## Ethics statement

This study does not fall under the scope of the Dutch Medical Research Involving Human Subjects Act (WMO). It therefore does not require approval from an accredited medical ethics committee in the Netherlands. However, in the UMC Utrecht, an independent quality check has been carried out to ensure compliance with legislation and regulations (regarding Informed Consent procedure, data management, privacy aspects and legal aspects). The research protocol for this study was registered under number 23U-0216.

## Results

### Selected children

**Figure 1.**
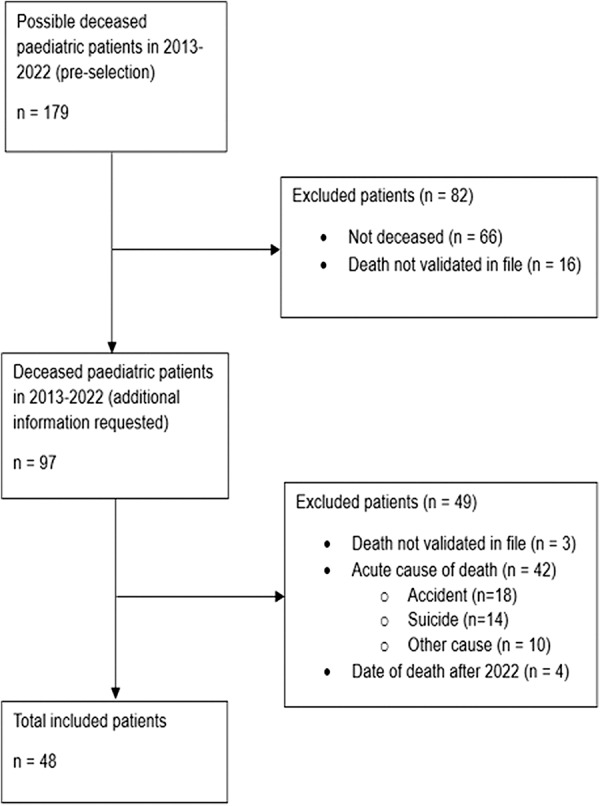
Process of patient selection.

In the first screening, in line with regulations for use of data from the JGPN database, limited data of the patient records of a total of 179 possible deceased children were extracted from the JGPN database. After screening on actual death of the child, 82 patients were excluded because they were not actually deceased or of whom death could not be confirmed. From the remaining 97 patients, based on additional requested data, another 42 patients were excluded because they died from an acute cause of death such as an accident or suicide. Three patients were excluded because their death could not be validated and four patients were excluded because their date of death was after 2022. Forty-eight children from 32 GP practices were included in the study. The process of patient selection is presented in Fig. 1.

### Missing data

There were no missing data for demographic characteristics. Several children had possibly missing data for hospital admissions. Data were extracted from a routine primary care existing data set, so it could be possible that more data are missing.

### Children characteristics

Median age of the children when they died was 10.0 years (Interquartile range [IQR] 1.5–17.1 years) and 56% were male. The most common main diagnoses were oncological (29%), congenital (29%), and metabolic conditions (23%). Survival after diagnosis was defined as the first time the diagnosis was documented by the GP until the registered date of death. Twelve out of 26 children (46%) from 0 to 2 years died in hospital compared to 4 out of 20 (20%) who died at home and 11 out of 26 children (42%) with congenital conditions died in hospital compared to 3 out of 20 (15%) who died at home. The shortest period of time living with the disease was 2 days and the longest period was 20 years and 7 months. Exploratory analysis identified cohorts based on the place of death of the child: children, who died in the hospital (*n* = 26, 54%), children who died at home (*n* = 20, 42%) and children for whom the place of death was unknown (*n* = 2, 4%) (Table 1).

**Table 1.** Children’ demographics, disease characteristics, by place of death

			Place of death		
		Total (*n* = 48) *n* (%)	Hospital (*n* = 26) *n* (%)	Home (*n* = 20) *n* (%)	Unknown (*n* = 2) *n* (%)
Sex	Male Female	27 (56.3) 21 (43.8)	13 (50.0) 13 (50.0)	12 (60.0) 8 (40.0)	2 (100.0) 0 (0.0)
Calendar age at death^a^	0–2 years 2–7 years 7–12 years 12 years and above	16 (33.3) 3 (6.3) 11 (22.9) 18 (37.5)	12 (46.2) 2 (7.7) 3 (11.5) 9 (34.6)	4 (20.0) 0 (0.0) 7 (35.0) 9 (45.0)	0 (0.0) 1 (50.0) 1 (50.0) 0 (0.0)
Calendar age at death (median, interquartile range [IQR])^b^		*M* = 10.0 years, (1.5–17.1)	*M* = 4.2 years, (1.0–15.3)	*M* = 11.0 years, (7.9–18.0)	*M* = 7.4 years Range = 5.3–9.4 years
Main diagnosis	Oncological condition Congenital condition Metabolic condition Other or unknown	14 (29.2) 14 (29.2) 11 (22.9) 9 (18.8)	5 (19.2) 11 (42.3) 5 (19.2) 5 (19.2)	9 (45.0) 3 (15.0) 5 (25.0) 3 (15.0)	0 (0.0) 0 (0.0) 1 (50.0) 1 (50.0)
Survival after diagnosis in years (median, interquartile range)		*M* = 1.2 years, (0.3–2.0)	*M* = 0.9 years, (0.2–4)	*M* = 1.4 years, (0.5–2.0)	*M* = 0.04 years, *Range* = <3 days–0.08 years
Survival after diagnosis in time categories^b^	0–6 months 6 months–1 year 1–2 years 2 or more years	17 (35.4) 4 (8.3) 16 (33.3) 11 (22.9)	10 (38.5) 3 (11.5) 6 (23.1) 7 (26.9)	5 (25.0) 1 (5.0) 10 (50.0) 4 (20.0)	2 (100.0) 0 (0.0) 0 (0.0) 0 (0.0)

Total percentages for characteristics may not equal 100 due to rounding.

a The age classification of children was based on the level of development adequate for the calendar age according to Piaget (Rabindran and Madanagopal 2020; Brunetta et al. 2022). These stages are: the sensorimotor stage (from 0 to 2 years old), the pre-operational stage (from 2 to 7 years old), the concrete operational stage (from 7 to 12 years old), and the formal operation stage (12 years and above).

b For pseudonomization of the deceased children, no birth date was available in the JGPN dataset for the researchers, but age at time of death in years, including two decimals.

### Number of GP consultations for children, GP home visits and unplanned hospital visits of children in the last three months of life

Forty-six out of 48 children (96%) had contact with their GP in the last 3 months (median 7 consultations), and 88% in the last month of life. Children had median 1.0 consultation in the third last month of life, 1.0 consultation in the second last month of life, and 3.0 consultations in the last month of life (Table 2).

**Table 2.** Number of GP consultations for children, GP home visits, care processes, and unplanned hospital visits in the last 3 months of life by place of death, as addressed in the medical record

	Time period	All children (*n* = 48)	Place of death hospital (*n* = 26)	Place of death home (*n* = 20)	*p*-Value*
**Number of GP consultations in the last 3 months of life**					
*GP consultations (home visits excluded)*	Third last month – median (IQR)	1.0 (0.0–3.0)	0.0 (0.0–1.0)	3.0 (2.0–4.0)	<0.001
	Second last month – median (IQR)	1.0 (0.0–4.0)	0.0 (0.0–3.3)	2.0 (1.0–5.0)	0.005
	Last month – median (IQR)	3.0 (2.0–6.0)	2.0 (1.0–4.0)	4.5 (2.3–8.8)	0.013
	Total in last 3 months – median (IQR)	7.0 (3.0–10.0)	3.5 (1.0–8.0)	9.5 (7.0–19.5)	<0.001
*GP home visits*	Third last month – median (IQR)	0.0 (0.0–0.0)	0.0 (0.0–0.0)	0.0 (0.0–0.8)	0.102
	Second last month – median (IQR)	0.0 (0.0–0.0)	0.0 (0.0–0.0)	0.0 (0.0–1.0)	0.048
	Last month – median (IQR)	0.0 (0.0–2.8)	0.0 (0.0–0.0)	3.0 (1.0–5.5)	<0.001
	Total – median (IQR)	0.5 (0.0–3.0)	0.0 (0.0–0.3)	3.0 (1.3–12.3)	<0.001
**Care processes**					*p*-Value*
Number of documented symptoms in the last 3 months at 3 time points	Third last month – median (IQR)^a^	1.0 (1.0–1.0)	1.0 (1.0–1.0)	1.0 (1.0–2.0)	0.114
	Last month – median (IQR)^b^	1.0 (1.0–1.0)	1.0 (1.0–1.0)	1.0 (1.0−2.0)	0.015
	Last 3 days – median (IQR)^c^	1.0 (1.0−2.8)	1.0 (1.0−1.0)	2.5 (1.0−4.0)	<0.001
					*p*-Value**
One or more medication prescriptions	Last 3 months – *n* (%)	40 (83.3)	18 (69.2)	20 (100.0)	0.017
One or more anticipatory medications prescription(s)	Last 3 months – *n* (%)	16 (33.3)	5 (19.2)	10 (50.0)	0.08
Documentation of start of terminal phase either written as terminal/dying phase/dying	*n* (%)	5 (10.4)	0 (0.0)	5 (10.4)	0.54
**Unplanned hospital visits in last 3 months of life**		**All children** **(*n* = 48)**	**Place of death hospital** **(*n* = 26)**	**Place of death home** **(*n* = 20)**	*p*-Value**
*1 or more contacts with GP out-of-hours service*	Last 3 months – *n* (%)	11 (22.9)	3 (11.5)	8 (40.0)	0.055
*1 or more emergency department visits*	Last 3 months – *n* (%)	14 (29.2)	10 (38.5)	3 (15.0)	0.178
1 or more hospital admissions	Last 3 months – *n* (%)	32 (66.7)	24 (92.3)	8 (40.0)	<0.001
		(*n* = 32)	(*n* = 24)	(*n* = 8)	*p*-Value*
Length of hospital admissions in days^d^	Last 3 months – median (IQR)	12.0 (5.3–27.0)	13.5 (6.3–31.5)	11.5 (2.3–14.0)	<0.001

* Mann-Whitney Test.

** Pearson’s chi-squared test.

a There was a wide range of symptoms and problems mentioned. Most frequently mentioned symptoms in the third last month were: dyspnea, pain, mucus secretion. At this time point for 5 children no symptoms were documented.

b There was a wide range of symptoms and problems mentioned. Most frequently mentioned symptoms in the last month were: dyspnea, pain, defecation problems. At this time point for 4 children no symptoms were documented.

c There was a wide range of symptoms and problems mentioned. Most frequently mentioned symptoms in the last 72 hours before death were: pain, fatigue, dyspnea, mucus secretion, drowsiness. At this time point for 3 children no symptoms were documented.

d The data for hospital admissions were not complete. For 6 out of 26 children who died in hospital, in the last month before death the admission date to hospital and discharge date (date of death) including length of last hospital admission were missing.

In the last 3 months, 26 children who died in hospital had median 3.5 GP consultations compared to median 9.5 GP consultations for 20 children who died at home (*p* < 0.001). The 26 children who died in hospital had median 0.0 GP home visits in the last three months of life compared to median 3.0 GP home visits for the 20 children who died at home (*p* < 0.001) (Table 2).

Fourteen children (29%) had at least one emergency department visit in the last 3 months of life, and 32 children (67%) were admitted to hospital at least once in the last 3 months of life. Median length of hospital admission for all children was 12.0 days (IQR 5.3–27.0) (Table 2).

### Identification of palliative care needs and involvement of other healthcare professionals

Seventeen (35%) children were documented by the GP as “having palliative care needs” or being in the palliative phase. For 12 children the GP had a personal conversation with the child and/or parents about the palliative identification. For the 5 remaining children the palliative intent was registered in the medical record, but no further conversation had been documented (Table 3).

**Table 3.** Information on identification of palliative care needs and involvement of other healthcare professionals by place of death, as addressed in the medical record

Items addressed in the medical record	Total (*n* = 48) *n* (%)	Place of death hospital (*n* = 26) *n* (%)	Place of death home (*n* = 20) *n* (%)
**Any identification of palliative care needs**			
GP had a conversation with patient/parents after palliative identification was conducted in hospital	10 (20.8)	3 (11.5)	7 (35.0)
GP had a conversation together with pediatrician at patient’s home	2 (4.2)	0 (0.0)	2 (10.0)
No GP conversation, identification only discussed in hospital (as documented in record)	3 (6.3)	0 (0.0)	3 (15.0)
No further conversation because of wish patient/parents (identification in hospital)	2 (4.2)	1 (3.8)	1 (5.0)
**Involvement of other healthcare professionals**			
Pediatrician or specialist in palliative care was involved	34 (70.8)	18 (69.2)	16 (80.0)
Pediatrician or specialist in palliative care initiated contact with GP	17 (35.4)^a^	4 (15.4)	13 (65.0)
Involved other healthcare professionals in the last 3 months of life			
Palliative (home care) team	7 (14.6)	1 (3.8)	6 (30.0)
Pediatric palliative care team	5 (10.4)	1 (3.8)	4 (20.0)
Pain team	6 (12.5)	1 (3.8)	5 (25.0)
Home care nurses	15 (31.3)	3 (11.5)	12 (60.0)

a Out of these 17 children, for 14 children the GP and pediatrician had 3 times or more contact about care issues, for 3 children the GP and pediatrician had 1 or 2 times contact in the last three months of life.

For 34 children (71%), it was documented that the pediatrician or other specialist was somehow involved in palliative care. The GP had an explicit request by the pediatrician or other specialist to collaborate or take the lead in palliative care for 17 children (35%) (Table 3). Whenever there was active engagement of pediatrician and GP, they maintained frequent (>3 times) contact in 14 (82%) cases (Table 3).

### Advance care planning

From all 48 children, 26 (54%) had some form of ACP conversations documented, mostly preferences for treatment and care. Most documented treatment and care preferences were: potential treatment limitation (77%), symptom control or comfort-care (58%), and Do-(Not-) Resuscitate (50%). Documentation of ACP conversations could either refer to a personal conversation between the GP and the child and family, or it could indicate that the agreements had been made in the hospital and communicated with the GP (Table 4).

**Table 4.** Extent to which advance care planning conversations and preferences regarding treatment and care were addressed in the medical record, by place of death

Items addressed	Total *n* = 48 *n* (%)	Place of death hospital (*n* = 26) n (%)	Place of death home (*n* = 20) *n* (%)
**Any information about advance care planning documented**	26 (54.2)	10 (38.5)	15 (75.0)
	*n* = 26 *n* (%)	*n* = 10 *n* (%)	*n* = 15 *n* (%)
*Discussion of preferences for treatment and care with child and/or family* If yes, items discussed^a^:			
Potential treatment limitation or discontinuation	20 (76.9)	8 (80.0)	12 (80.0)
Symptom control/comfort care	15 (57.7)	5 (50.0)	10 (66.7)
Do-(Not-) Resuscitate	13 (50.0)	3 (30.0)	10 (66.7)
Hospital admissions	7 (26.9)	1 (10.0)	6 (40.0)
No artificial respiration	7 (26.9)	4 (40.0)	3 (20.0)
Palliative sedation	5 (19.2)	0 (0.0)	5 (33.3)
Euthanasia	4 (15.4)	0 (0.0)	4 (26.7)
Other	5 (19.2)	3 (30.0)	1 (6.7)
*Time between diagnosis and first ACP documentation in years (median, [IQR])*	1.07, (13 days to 1.9 years)	0.41, (7 days to 3.3 years)	1.2 years, (15 days to 1.7 years)
∘ Less than 1 month^b^ – *n* (%)	9 (34.6)	3 (30.0)	5 (33.3)
1 month to 1 year – *n* (%)	3 (11.5)	3 (30.0)	0 (0.0)
1 to 2 years – *n* (%)	8 (30.7)	1 (10.0)	7 (46.7)
2 years and more – n (%)	6 (23.1)	3 (30.0)	3 (20.0)
*Preferred place of death addressed*	4 (8.3)	1 (10.0)	3 (30.0)

a Multiple answers possible.

b The date of diagnosis was the date of the main diagnose. For children with congenital conditions, the registration date to the general practice was used as date of diagnosis.

The median time from diagnosis until the first ACP conversation was 1 year and 25 days (IQR 13 days to 1.9 years). For 9 children (35%) the conversation took place in less than 4 weeks after diagnosis. For 14 children (54%) the first time the topic was discussed was over a year after diagnosis (Table 4).

For only 4 out of 48 children their preferred place of death was documented in the GP record and those 4 children died at their preferred place of death (3 at home, 1 at the hospital).

## Discussion

### Summary

The two categories of patients (death at home or in hospital) in our study are mainly distinguished by age and type of pathology. The results of this study demonstrate that GPs are highly involved in providing care for children at the end of life and their families. Children had median 7.0 GP consultations and median 0.5 GP home visits in the last 3 months of their life. However, the palliative nature of these children and ACP discussions are not routinely documented and/or performed by GPs. For more than two third of the children it was documented that a pediatrician was involved, but for only about one third of the children was it documented that the pediatrician or specialist in palliative care and GP communicated with each other. In cases where the GP was actively involved by the pediatrician, they contacted each other regularly regarding ongoing care issues.

### Comparison with existing literature

#### GPs providing many GP consultations to relatively low numbers of children in need of palliative care in their practice

The majority of pediatric deaths per year in the Netherlands occur in children under the age of 1 (CBS StatLine 2024) and the number of deaths rises again from the age of 15 onwards. Most of these children under the age of 1 die from a congenital disease (CBS StatLine 2024). The results of this study show that, probably due to the rareness of these diseases, the most frequent main diagnosis of children who died in hospital was a congenital disease.

It is known that most children with a life-limiting illness and their parents prefer to stay at home during the palliative phase (Vickers et al. 2007; Van der Geest et al. 2016). Advantages mentioned for the child and family are privacy, the presence of siblings, and parents feeling in control (Hynson et al. 2003). In the home setting GPs play an important role in providing palliative care for both adult patients (Grant et al. 2024b) and children (Van der Geest et al. 2016, 2017; Mitchell et al. 2021). However, studies show that GPs are regularly confronted with palliative care for adult patients (Rhee et al. 2020; Grant et al. 2024a, 2024b), but most GPs rarely provide palliative care to children (Van der Geest et al. 2016).

The results of our study confirm that the number of children that GPs provide palliative care for was relatively small, with 48 patients identified in a 10-year period for approximately 200 GPs. Yet this care provision was intense, especially for children who died at home, in the last 3 months of life the median GP consultations and median GP visits were high compared to the median 4 GP consultations (including GP visits) for adult cancer patients in the last three months of life that was found by Grant et al. (2024a). For the children who died at home, GPs were highly involved, with the patients accessing GP care median 4.5 times in the last month of life, and increasing contacts over the last 3 months (including home visits). Care provision also peaked in the last month of life, likely a reflection of increasing care needs, and GPs possibly responding appropriately to these needs, although we do not know if there were any care needs due to a lack of adequate care by the GP or other healthcare professionals. It would not be surprising if palliative care provided by GPs for these children is not always optimal. It is well known that caring for these children is challenging for many GPs. Reasons for this are specific aspects of pediatric palliative care compared to adult palliative care as mentioned in the literature: the developmental stage of the child has consequences for GP communication about death and dying, and psychological factors are different for children than for adult patients (Van der Geest et al. 2016; Vallianatos et al. 2021). Another reason may be that, in the Netherlands, palliative care is not a medical specialty, and specialist community palliative care access is limited or not available. This results in GPs being largely responsible for community palliative care provision, often with limited support, thus emphasizing the need for support for GPs providing palliative care to patients with complex care needs, such as pediatric patients. GPs are also expected to provide bereavement care to the family after the death of the child (Dutch Association for Pediatrics [NVK] 2022), but our study did not provide any insight into the actually provided bereavement care by the GP to the family. Our findings raise the question if and if so, how GPs could be better supported in providing palliative care to children. Besides basic training a structure of adequate resources for expert advice seems necessary (Van der Geest et al. 2016; Mitchell et al. 2020).

Several studies on GP experiences with palliative pediatric care have been conducted (Straatman and Miller 2013; Neilson et al. 2015; Van der Geest et al. 2017), but to our best knowledge there are very few studies on documentation of actual palliative care provision for children in GP practice. Jarvis et al. (2020) found an association between GP attendance patterns and use of urgent hospital care for children and young people with a life-limiting condition residing at home in England, with less regular GP attendance associated with higher urgent hospital care (Jarvis et al. 2020).

### No routinely documentation of advance care planning information

We identified that information on ACP was not routinely documented. In the medical records, there was little information on preferences for treatment and care, which may make it more complicated for all involved care professionals in the GP clinic to provide personalized palliative care to the children and their families. For children with life-limiting or life-threatening diseases, parents aim for integrated care including both control of the disease and symptom management, as well as quality of life for their seriously ill child and their family as a whole (Verberne et al. 2017). Also in pediatric palliative care, ACP exploring the child and family values, goals, and preferences is essential (Rietjens et al. 2017). Most professionals in pediatric palliative care acknowledge this importance of ACP, but many barriers for conducting ACP conversations with parents and children still exist (Lotz et al. 2015; Sanderson et al. 2016; Engel et al. 2024). In 2020, Fahner et al. found that ACP documentation in pediatric palliative was insufficient according to 49% of 207 pediatricians who responded to a survey asking for their experiences with ACP (Fahner et al. 2020). In our study, for almost three quarters of children a pediatrician or specialist in palliative care was involved. It is possible that the pediatrician or specialist in palliative care conducted ACP conversations, but that this was not well documented in the GP record. However, the lack of ACP documentation may also be related to the fact that the child and family did not have an ACP conversation at all, possibly due to the challenging nature of engaging with young children and their families around death and dying or an unclear division of responsibilities regarding ACP between specialists and GPs. This lack of ACP and ACP documentation was also found in other studies on the prevalence and characteristics of ACP (Meeussen et al. 2011; Durall et al. 2012; Fahner et al. 2020).

## Strengths and limitations

A strength of this study is that it is one of the few studies in which documentation of pediatric palliative care by GPs was studied in routine primary care data from medical records of deceased children. Another strength is that regarding place of death, over half of the children who received GP care died in hospital and most other children died at home. Therefore, we compared GP care between the two groups, although we realize the methodological limitation of comparing these two groups because we determined this independent variable only after the dependent variable had appeared. Limitations are that it was a very small group of children that nonetheless represents actual pediatric care practice, and that we had to do research in a dataset in which no hospital letters were available. We may have missed information that was communicated or transferred between the pediatrician or other healthcare professionals and GP but not documented in the medical record. Because of the small group of children, caution is advised in drawing strong conclusions from the statistical differences. We expect that our findings are representative for the Netherlands and possibly for other countries within and outside Europe, although caution is advised because of differences in health care systems and in the role of the GP.

### Need for good collaboration among involved healthcare professionals and support from specialized professionals

GPs should have knowledge of providing (pediatric) palliative care at home. Training of all GPs in complex and highly variable situations of pediatric palliative care seems not very efficient. Many of the skills needed for pediatric palliative care are already part of the GP toolkit (i.e. symptom management, ACP discussions, care for the family, care in the home setting) (Dutch Association for Pediatrics [NVK] 2022). Therefore, good collaboration among healthcare professionals is essential to provide high-quality palliative care. Support from specialized clinicians and consultation-based contact with a (specialized) hospital-based palliative care team that is also available during out-of-hours may be an efficient approach (Van der Geest et al. 2016; Mitchell et al. 2020; Vallianatos et al. 2021).

In countries such as the Netherlands, the health care system is increasingly focused on providing care in the home setting. While this focus is primarily driven by an aging population with increasing rates of chronic diseases, it is a transition that will also impact the delivery of pediatric care, particularly palliative care. The home environment is ideally situated to provide end of life care for these children, where they can be surrounded and supported by their parents, siblings, and friends. Yet, the quality of this care is dependent upon local general practice services, to provide appropriate, accessible and timely care for the child and their family network (Mitchell et al. 2020). Specialist pediatric palliative care services may be an important element of this care, but will not always be readily accessible (particularly in rural regions), may have limited integration with local formal and informal care services, and are limited to provide ongoing care for the family members through the palliative trajectory and after the death of the child.

In conclusion, these data demonstrate that while GPs rarely provide palliative care for pediatric patients, that when they do so, they are intensely involved in this care, and are one of the key facilitators, together with pediatricians, home care nurses, and palliative care specialists, to support pediatric patients and their families in the home environment. The palliative nature of these children and ACP discussions are not routinely documented and/or performed by GPs. Further insights into guidance that supports GPs, in collaboration with other healthcare professionals, in providing care for children in need of palliative care at home and their families are needed.

## Supporting information

10.1017/S1478951526102077.sm001 Engel et al. supplementary material 1 Engel et al. supplementary material

10.1017/S1478951526102077.sm002 Engel et al. supplementary material 2 Engel et al. supplementary material

## Supplementary material

The supplementary material for this article can be found at https://doi.org/10.1017/S1478951526102077.
