# Supplementary material for: Palliative care provision for children in general practice: A retrospective cohort study
Source: Palliat Support Care. 2026 Mar 26;24:e88. doi: 10.1017/S1478951526102077 (PMC13166309; doi:10.1017/S1478951526102077)
Supplement: Engel et al. supplementary material 2 — Engel et al. supplementary material [file S1478951526102077sup002.docx]

**Questionnaire**

**Palliative care provision for children in general practice: a retrospective cohort study**

*Child characteristics*

- - - 1. Age at the time of death (year and month)

1. Sex of child
2. Are there other people registered at their address / or in notes (SOEP lines)

*Death*

1. Date of death
   - Is this date validated in the medical notes?
2. Place of death
3. Major disease causing death
   - Major diagnosis
   - How far prior to death was this diagnosis known (not known, days, weeks, 1-3 months, 4-8 months, 9-12months, years)
   - Describe what factors caused death (ie. pneumonia)
   - Major comorbidities
4. Other diagnoses impacting death
5. Is a caregiver(s) listed in the notes?
6. Was death sudden or unexpected?
   - If death was due to an acute AND unexpected cause (ie. car accident) - no further information will be collected.
7. Which healthcare professionals and other caregivers were documented in the last 3 months of life?
   - Which healthcare professionals and other caregivers were listed in the child file
   - Which care organisations were involved in the last year of life.

*Identification*

1. Was the child noted to be palliative / or have palliative intent of treatment?

- When was this first noted? - date
- If yes, was the palliative intent of treatment discussed with the child / or family?

*Advance care palling discussions*

1. Did advance care planning discussions occur?

- When were these first discussed?
- Record all information indicating that the following has been discussed with the child and/or family
  - Advance Care Planning in the context of agreements on care and treatment
  - Preferred care and treatment in a broad sense
- Which treatment decisions were discussed? (hospital admission, Do-(Not-)Resuscitate, artificial respiration, limitation to medication treatment, symptom control/comfort care, palliative sedation, euthanasia)
  - If euthanasia was discussed, did this occur?
- Was an advance care plan and/or advance care directives formulated?
- Was the preferred place of death documented?

*Place of death*

1. Where did the child die? (hospital, home, other)
2. Was there preferred place of death discussed?
3. Did they die in this place?

*Symptoms*

1. Which symptoms were noted?
   - All documented symptoms in the last 3 months of life at 3 time points (in the third last month, in the last month, in the last 72 hours before death)

*Hospital and GP out-of-hours service use*

1. Did the child contact the GP out-of-hours service in the last 3 months?
   - What were the dates of these consultations
   - For what reasons did they contact the GP out-of-hours service
   - Were any of these contacts related to terminal care
     1. If YES, please describe what management was provided (ie. starting pomp, titrating medications).
2. Did the child visit the emergency department in the last 3 months?
   - What were the dates of these visits
   - Were they admitted on any of these visits
   - What were the indications
3. Was the child admitted to hospital in the last 3 months?
   - What were the dates of these admissions
   - What were the indications for admission
   - Did discharge summaries contain information about prognosis, advanced care planning information, is clear who is the leading physician

*Consultations*

1. Number of consultations in last 3 months (in third last month of life, in second last month, in last month)
   - Number of consultations
   - Number of home visits
   - Main reasons for home visits
   - Number of administrative notes
   - Did paediatrician (or other hospital physician) actively involve the GP in the palliative care trajectory
     1. If YES, number of contacts between paediatrician and GP

*Medication use*

1. Medications used in the 3 months prior to death
2. *W*ere the child’s medications reviewed and/or ceased in the period leading up to their death?
   - Which medications were stopped and when

*Terminal care at home*

1. Did the child have terminal care at home (if Yes – continue, if No- end of questionnaire)
2. What medications was the child using prior to terminal phase
   - Type and dose of medication
   - Were there issues reported relating to accessing medications
3. Were anticipatory medications prescribed?
   - Which medications – type and dose
   - When were these prescribed
   - Were these medications used and when
4. Was a catheter used?
   - Were there any issues relating to this
5. Were there any other devices used? Or required?
   - If so describe
6. Did the child have an implantable defibrillator.
   - If yes, when was this deactivated, and were there issues with this
7. Any other issues / challenges in the terminal phase?
8. Did the GP provide terminal care after hours (ie. notes describing an after hours phone call or visit)
